# Supplementary material for: Age-Dependent Pleomorphism in Mycobacterium monacense Cultures
Source: Microorganisms. 2025 Feb 20;13(3):475. doi: 10.3390/microorganisms13030475 (PMC11946739; doi:10.3390/microorganisms13030475)

Supplementary material

Age-dependent pleomorphism in *Mycobacterium monacense* cultures

Malavika Ramesh, Phani Rama Krishna Behra, B. M. Fredrik Pettersson,

Santanu Dasgupta and Leif A. Kirsebom*

Department of Cell and Molecular Biology
Box 596, Biomedical Centre
SE-751 24 Uppsala, Sweden

*Corresponding author
Tel no +46 18 471 4068
Fax no +46 18 53 03 96
Email Leif.Kirsebom@icm.uu.se

Supplementary material

Supplementary Table S1: Compilation of primers and probes used in this study.

Supplementary Table S2: Compilation of the strains used in this work with their corresponding nomenclature.

Supplementary Table S3: Generation times (GT) for *Mmon*^{RFPHyg} grown in different media and conditions as described in Section 2.

Supplementary Table S4: Compilation of mRNA levels for selected genes in *Mmon*^{RFPHyg} at three different time points (6 days, 14 days and 48 days) relative to the levels in exponentially growing cells (3 days time point). The values are given as log_2-fold changes.

Supplementary Table S5: Compilation of predicted adenylate cyclase genes with their locus tags in *Mmon*^{T} and *Mmar*^{T}.

Supplementary Figures S1-S12

Supplementary results

Identification of putative promoters, sigma factors and regulatory "elements"

To address whether the regulation of *dnaK*_3 expression in *Mmon* and *Mmar* is different, we decided, as a first step, to identify sigma factors involved in transcription of the *dnaK*_3 gene in *Mmon*^{T} and *Mmar*^{T}. Hence, we compared the upstream regions (300 bps) of the *dnaK*_3^{Mmon}, *dnaK*_3^{Mmar} and *dnaK*^{MtbH37Rv} coding sequences to identify putative sigma factor binding regions. First, SigH was reported to regulate the expression of *dnaK* in *Mtb* [102]. On the basis of this and sequence similarity comparing the upstream regions of *dnaK*_3 *Mmon*^{T}, *Mmar*^{T} and *Mtb*^{H37Rv} we predict that SigH is also instrumental in regulating *dnaK*_3 expression in both *Mmon*^{T} and *Mmar*^{T} (referred to as SigH^{P1}; Fig S7).

Using consensus sequences recognized by different sigma factors in *Mtb*^{H37Rv} [142], we identified an additional putative SigH promoter (SigH^{P2}) as well as promoters for SigE (SigE^{P1}

and SigE^{P2}), SigG, and SigM in *Mmon*^{T}. The predicted SigE^{P1} and SigG promoters overlap with SigH^{P1} (Fig S7) while for the SigE^{P1}, SigE^{P2} and SigH^{P2} -10 promoter sequences four (of five) nucleotides match the *Mtb* consensus sequence. For the -35 box we also noted that there are differences relative to the *Mtb* consensus sequences. We also identified a SigB -10 and two possible upstream -35 boxes. Together this indicates that transcriptional control of *dnaK*_3^{Mmon} might involve several sigma factors SigE, SigG, SigH and SigM and possibly also SigB.

For *Mmar*^{T}, we identified SigD, SigE, and SigH putative promoters upstream of *dnaK*_3^{Mmar} in addition to SigH^{P1} (see above) where SigE^{P1} overlap with SigH^{P1}. As in the case of *dnaK*_3^{Mmon}, SigE^{P2} and SigH^{P2} also overlap in *dnaK*_3^{Mmar} (Fig S7; we noted that there is a four-nucleotides match out of five in the -10 box, compared to the *Mtb* consensus sequence for SigH^{P1} and SigE^{P1}). Moreover, the "SigE^{P1}-SigH^{P1}" overlap with a putative "SigD -10 box" but no -35 box could be identified (Fig S7) leading to the question whether SigD is involved in transcribing *dnaK*_3^{Mmar}. A third putative SigH promoter (SigH^{P3}) just upstream of the -35 box of SigE^{P2}/SigH^{P2} was also identified. Together, this suggested that *dnaK*_3 expression in both *Mmon* and *Mmar* is probably dictated by SigE and SigH. Moreover, we note other differences such as the presence of putative SigG and SigM promoters that might have an impact on the expression of *dnaK*_3 in *Mmon*^{T}.

We also predicted other regulatory regions upstream of *dnaK*_3 (Fig S7). As in *Mtb*, sequence alignment suggested that the promoter regions of both *Mmon*^{T} and *Mmar*^{T} encompass two putative binding sites for the transcription repressor HspR, HAIR (HspR associated inverted repeat [99]) and two CRP binding sites (catabolite repressor protein) [103,104]. Given that CRP binds cAMP and influences the promoter activity, we predicted the presence of 14 and 24 adenylate cyclase genes in *Mmon*^{T} and *Mmar*^{T}, respectively. Among these, nine represent homologous genes present in these two mycobacteria (Table S5). Whether the level of cAMP influence CRP binding and *dnaK*_3 transcription remains to be determined. For *Mmon*^{T}, we also

identified putative RegX3 binding site in the *dnaK*_3 upstream region [104] and two *regX*3 genes (46% identity at protein level). Moreover, we also identified a putative MtrA binding site in the *Mmar*^{T} *dnaK*_3 region [143], which could not be detected in *Mmon*^{T} (Fig S7). Therefore, binding of MtrA might play a role in this context but this requires further investigation.

Taken together, these findings emphasize similarities and more importantly, differences that might explain the divergent expression of *dnaK*_3 in *Mmon*^{T} and *Mmar*^{T} (see Section 4).

Analysis of sigma factor mRNA levels at different growth stages

Mmon^T has 21 sigma factor genes with three categorized as *sigF*, two as *sigH*, three as *sigJ* and two as *sigL*. The remaining sigma factors were classified to originate from single genes while the 17 *Mmar*^{T} sigma factors are classified and transcribed from single genes with the exception of *sigC*, which exists in two copies (Fig S9; see also [41]). It is also noteworthy that *Mmon*^{RFPHyg} SigB and SigE mRNA levels in exponentially growing cells (three days) are high, ≈45% of the total sigma factor transcripts. The fractions of SigB and SigE mRNAs increased only slightly (to ≈55%) upon ageing. For SigH1, the level decreased with time and varied between ≈4 - 8% while the fraction of SigH2 mRNA was ≤0.1% irrespective of "age". This is in contrast to *Mmar*^{RFPHyg} (a derivative of *Mmar*^{T} with *rfp* and *hyg*^{R} inserted into the *attB* site) [41] where both SigB and SigE mRNA levels increased in stationary phase relative to the levels detected in exponentially growing cells while the single SigH gene transcript level did not change in stationary cells (Fig S9H-J). However, compared to the other sigma factors, the SigH mRNA level is ≈5% in exponential growing *Mmar*^{RFPHyg} cells and it decreases in stationary phase (Fig S9H-J), see also Refs [41,67]. Noteworthy, for some of the other *Mmon*^{RFPHyg} ECF sigma factor mRNAs, such as SigD and SigF3, the fraction of the mRNA levels relative to the total fraction of sigma factor mRNA transcripts decrease upon ageing. We also note that in particular the SigL2

increases while SigF2 decreases comparing exponentially (3 days) growing cells and 48 days old cells (Fig 9C-G).

Endospore staining (Schaeffer-Fulton Stain)

The Schaeffer-Fulton staining [144,145] differentiates between endospores and vegetative cells. Briefly, malachite green was used as a primary stain and after washing while Safranin was used as a counter-stain that stains vegetative cells red. Technically, spores should appear green due to malachite green staining while the vegetative cells, which have lost the cell membrane integrity (due to the heating steps), take up the safranin after washing and appear red when viewed under the microscope. Endospores appear yellow or pink. The refractive cells appeared both red and green (yellow when overlaying fields) suggesting that the refractive cells could be endospores (Figure S11). *Mmon* cells were, however, sensitive to wet heat and did not show any Dipicolinic acid content (see main text).

Supplementary references 142-157, see Reference list in the main text.

Table S1

Primers and Probes	
16s Forward	GCTGCCTYCCGTAGGAGTYTGG
16s Reverse	RAATTGACGGGGGCCCGCACAAC
dnaK3-Mmon_Forward	TTTTCTTAAGTGAGTACCACCGAAGGGGTGTATAT<br>GGCTCGTGCGGTCGGAATC
dnaK3-Mmon_Reverse	TTACTAGTTCGAGCGCCTCGCGTGAGCGAGGCG<br>CTCGATCACTTGTTCTCCCGGTCATCGT
dnaK3-Mmon-antisense_Forward	TTTTCTTAAGTCACTTGTTCTCCCGGTCATCGT
dnaK3-Mmon-antisense_Reverse	TTACTAGTTCGAGCGCCTCGCGTGAGCGAGGCG<br>CTCGAAGGAGGACAAACCATGGCTCGTGCG
pMIND01_Forward (pBS401 screening)	CATCGATAACTTTATCTTAGATAAAGTGACTGC
FP10109_Reverse (pBS401 screening)	GAGCGAGGAAGCGGAAGAGC
dnaK3_probe	6FAM-CTGCCCTACATCACC-MGBNFQ
dnaK3_Forward (specific to dnaK3-Mmon)	AGCCAGAGCACCTCGATCA
dnaK3_Reverse (specific to dnaK3-Mmon)	TCGAGGAACAGCGGGTTCT
16S rRNA_UL051119 rrs	GGTGGTTTGTCGCGTTGTTC
16S rRNA_UL051120 rrs	GCCCGCACGCTCACA
16S probe_UL051114 rrs	6FAM-TGAAATCTCACGGCTTAA-MGBNFQ
MMAR_0637 dnaK Fw (specific to Mmar)	CGCAGAGCACCTCGATCAA
MMAR_0637 dnaK Rv (specific to Mmar)	CGAGGAACAGCGGGTTCTT

6-FAM= 6-carboxyfluorescein MGBNFQ= Minor Groove Binder Non-Fluorescent Quencher

Table S2

**Strain nomenclature**	**Strain used**	**Plasmid**
Mmon^{T}	*M. monacense* DSM44395	No plasmid
Mmon^{RFPHyg}	*M. monacense* DSM44395+ pDEAM5	pDEAM5 (RFP, HygR, integrative)
Mmon^{RFPKan}	*M. monacense* DSM44395+ pDEAM2	pDEAM2 (RFP, KanR integrative)
Mmon^{pBS401}	*M. monacense* DSM44395+ pBS401	pBS401 (HygR, non-integrative)
Mboe^{T}	*M. boenickei* DSM44677	No plasmid
Mmar^{T}	*M. marinum* CCUG20998	No plasmid
Mmar^{RFPHyg}	*M. marinum* CCUG20998+ pDEAM5	pDEAM5 (RFP, HygR, integrative)
Mmar^{pBS401}	*M. mar*^{T}+pBS401	pBS401 (HygR, non-integrative)
Mmar$^{pBS401\text{-}dnaK3Mmar}$	*M. mar*^{T}+pBS401-*dnaK3*Mmar	pBS401+ *dnaK3* (HygR, non-integrative)
Mmar$^{pBS401\text{-}dnaK3Mmon}$	*M. mar*^{T}+pBS401-*dnaK3*Mmon	pBS401+ *dnaK3* (HygR, non-integrative)
Mmar$^{pBS401\text{-}(Anti)dnaK3}$	*M. mar*^{T}+pBS401-(Anti)*dnaK3*Mmon	pBS401+ (Anti)*dnaK3* (HygR, non-integrative)
Mmar^{pIGn}	*M. mar*^{T}+pIGn-*dnaK3*Mmon	pIGn (HygR, non-integrative with *lacZ* gene)
Mmar$^{pIGn\text{-}dnaK3Mmon}$	*M. mar*^{T}+pIGn-*dnaK3*Mmon	pIGn+ *dnaK3* (HygR, non-integrative with *lacZ* fusion to *dnaK3*)
Mmar$^{pIGn\text{-}dnaK3Mmar}$	*M. mar*^{T}+pIGn-*dnaK3*Mmar	pIGn+ *dnaK3* (HygR, non-integrative with *lacZ* fusion to *dnaK3*)

Table S3

	Original	Re-suspended	Glycerol	Tween	Expo II cells				Expo I cells	
					Fresh (u.d)	Fresh (1000 fold)	Spent (u.d)	Spent (1000 fold)	Fresh (u.d)	Spent (u.d)
Phase I	5±0.44	N.A	N.A	N.A	16±0.69	7±1.4	14±4.2	8±1.0	7±0.3	4±0.9
Phase II	21±0.98	20±0.6	18±1.1	14±1	35±8.2	20±1.4	31±5.4	32±7.7	20±7.0	20±1.7
Phase III	34±9.3		42±1.5	53±2						

Table S4

Gene name	Established function	Gene in *M. monacense*	Locus tag	Differential expression 6d/3d	14d/3d	48d/3d
		**Peptidoglycan**				
mur gene cluster	ATP dependant ligases, synthetases involved in PG biosynthesis [146]	*murA*	MMON_03992c	-0.45	-0.36	-1.22
		murB	MMON_00689	0.42	0.14	0.23
		murC	MMON_03394c	0.32	-0.83	-0.49
		murD	MMON_03397c	0.08	-1.26	-1.04
		*murE*_1	MMON_03400c	-0.18	-0.64	-0.53
		*murE*_2	MMON_05317	0.14	0.49	-0.13
		murF	MMON_03399c	0.01	-1.33	-1.15
		murG	MMON_03395c	0.23	-0.8	0.03
		murI	MMON_03964c	0.28	-0.34	0.43
ftsW	PG biosynthesis ; septum formation [72,147,148]	*ftsW*_1	MMON_00033c	0.28	-0.51	-0.66
pbp gene cluster	PG biosynthesis ; septum formation [72,147,148,149]	*pbpA*_1	MMON_00032c	0.12	-0.36	-1.1
		*pbpA*_2	MMON_05372	0.47	0.26	0.72
		pbpB	MMON_03401c	0.53	-1.14	-0.72
		pbpI	MMON_02096	-0.11	-1.66	-0.68
		pbpX	MMON_04832c	0.45	0.43	0.51
		**Divisome & Elongation Complexes**				
pknA-*pknB*	Cell shape regulation [70]	*pknA*	MMON_00031c	-0.21	-1.16	-0.96
		pknB	MMON_00030c	0.14	-0.83	-0.36
ripA (Divisome)	PG hydrolase, cysteine protease- cell wall synthesis & cell div [150-152].	*ripA*_1	MMON_02512	-0.02	-2.27	-1.92
		*ripA*_2	MMON_02710c	-0.25	0.31	-0.61
		*ripA*_3	MMON_02861c	0.14	-0.61	-2.52
cwsA (Elongation and Divisome)	PG synthesis & cell shape maintenance [75,149]	*cwsA*	MMON_00024c	-0.02	-0.27	1.23
ponA (Elongation and Divisome)	Codes for penicillin binding protein1 - PBP1 [73,153]	*ponA1*_1	MMON_01520	0.63	2.64	2.27
		*ponA1*_2	MMON_05242	-0.35	-0.52	-0.05
		*ponA1*_3	MMON_05796	-0.15	-1.07	-1
crgA (Divisome)	PG synthesis & cell shape maintenance [75,149]	*crgA*	MMON_00027c	-0.21	-0.32	-0.52
wag31 (Divisome)	Cell shape regulation [70,154]	*wag31*	MMON_01995	0.11	-0.83	-0.89
ftsZ (Divisome)	Z-ring assembly and cell division [154,155]	*ftsZ*	MMON_03392	0.17	-2	-2.37
		**Arabinogalactan**				
fbp gene cluster	Cell wall synthesis & cell division [156]	*fbpA*_1	MMON_02030c	-0.6	-3.01	-1.63
		*fbpA*_2	MMON_02726c	-0.22	0	-0.45
cop1	Mycolic acid synthesis in *C. glutamicum* [69]	*fbpC*_3	MMON_02961	1.56	1.27	0.14

		*fbpC*_4	MMON_04544c	1.13	0.85	1.3
		*fbpC2*_1	MMON_02724c	0.02	1.64	1.84
		*fbpC2*_2	MMON_02986c	0.15	-0.68	0.47
dprE	Cell wall arabinogalactan synthesis pathway [160]	*dprE1*_1	MMON_05323	0.39	2.46	2.01
		*dprE1*_2	MMON_05421	-0.21	-1.53	-0.5
**Others**						
dnaK	Heat-shock protein (Hsp70)	*dnaK*_1	MMON_00422	-0.3	-0.73	-0.93
		*dnaK*_2	MMON_00478c	-0.02	-0.74	-0.55
		*dnaK*_3	MMON_00498	0.31	2.46	4.42
		*dnaK*_4	MMON_03526c	-0.26	0.05	-0.73
grpE	Nucleotide exchange protein	*grpE*	MMON_00499	0.52	2.6	4.61
dnaJ	Heat shock protein/ Chaperone	*dnaJ*_1	MMON_00500	0.69	2.44	4.45
hspR	Heat shock protein regulator	*hspR*	MMON_00501	-0.13	1.28	3.82
**Other Serine/Threonine Kinases**						
pknB	Ser-Thr Kinases	*pknB*_2	MMON_03095	-0.07	-1.83	-2.21
		*pknB*_3	MMON_05819	0.5	-1.2	-2.88
pknE	Ser-Thr Kinases	*pknE*_1	MMON_00792	0.23	0.67	-0.31
		*pknE*_2	MMON_01989	-0.16	-0.74	-0.27
pknF	Ser-Thr Kinases	*pknF*_1	MMON_00835	0.45	-0.75	-2.68
		*pknF*_2	MMON_04694	-0.1	-0.32	-0.42
		*pknF*_3	MMON_05428	0.34	0.43	0.12
pknG	Ser-Thr Kinases	*pknG*	MMON_00574	0.21	0.58	-0.16
pknH	Ser-Thr Kinases	*pknH*_1	MMON_00265	0.23	-0.12	0.95
		*pknH*_2	MMON_00822	-0.26	0.34	-0.52
		*pknH*_3	MMON_03372	0.28	1.81	-1.46
		*pknH*_4	MMON_03540	0.27	-0.3	-1.48
		*pknH*_5	MMON_03634	0.75	2.84	1.87
pknK	Ser-Thr Kinases	*pknK*	MMON_04725	0.45	1.69	2.23
pknL	Ser-Thr Kinases	*pknL*	MMON_03411	-0.35	0.04	0.23
prkC	Ser-Thr Kinases	*prkC*	MMON_05261	0.16	-0.25	-0.76

Table S5

M. marinum CCUG 20998		Gene_product	*M. monacense* DSM44395	
Locus_tag	Gene_symbol		Locus_tag	Gene_symbol
CCUG_00147		pH-sensitive adenylate cyclase		
CCUG_00617	*cya*_1	Adenylate cyclase		
CCUG_00678	*cyaA*_1	Adenylate cyclase 1		
CCUG_00879	*cyaB*_1	Adenylate cyclase 2		
CCUG_02328	*cya*_2	Adenylate cyclase	MMON_03208	*cya*
CCUG_02355		pH-sensitive adenylate cyclase	MMON_03152	
CCUG_02436	*cyaB*_2	Adenylate cyclase 2		
CCUG_02933	*cyaA*_2	Adenylate cyclase 1		
CCUG_02934	*cyaA*_3	Adenylate cyclase 1	MMON_01322	*cyaA*_1
CCUG_03136		pH-sensitive adenylate cyclase		
CCUG_03507	*cyaB*_3	Adenylate cyclase 2		
CCUG_03700	*cya*_3	Adenylate cyclase		
CCUG_03702	*cya*_4	Adenylate cyclase		
CCUG_03726	*cyaB*_4	Adenylate cyclase 2		
CCUG_04032	*cyaB*_5	Adenylate cyclase 2	MMON_03985	*cyaB*_4
CCUG_04033	*cyaB*_6	Adenylate cyclase 2		
CCUG_04081	*cyaB*_7	Adenylate cyclase 2		
CCUG_04135		pH-sensitive adenylate cyclase	MMON_04099	
CCUG_04321	*cyaA*_4	Adenylate cyclase 1	MMON_04462*	
CCUG_04352	*cyaA*_5	Adenylate cyclase 1	MMON_03946	*cyaA*_3
CCUG_04416	*cyaB*_8	Adenylate cyclase 2		
CCUG_05125	*cyaB*_9	Adenylate cyclase 2	MMON_05210	*cyaB*_5
CCUG_05244	*cyaA*_6	Adenylate cyclase 1		
CCUG_05247	*cyaB*_10	Adenylate cyclase 2	MMON_01321	*cyaB*_3
		Adenylate cyclase 2	MMON_01319	*cyaB*_1
		Adenylate cyclase 2	MMON_01320	*cyaB*_2
		pH-sensitive adenylate cyclase	MMON_01581	
		Adenylate cyclase 1	MMON_02377	*cyaA*_2
		pH-sensitive adenylate cyclase	MMON_02696	

*A homologue of CCUG_04321 coding for pH-sensitive adenylate cyclase

Supplementary Figures and legends

Figure S1

Colony morphology of *Mmon*^{T} on different media.

Appearance of smooth, yellow colony morphology of *Mmon*^{T} after incubation on 7H10, LA and mG media for 15 days at 37°C as indicated. The yellow pigmentation seems to show small variation between the different media albeit the colony morphology appears smooth irrespective of media.

Fig S1

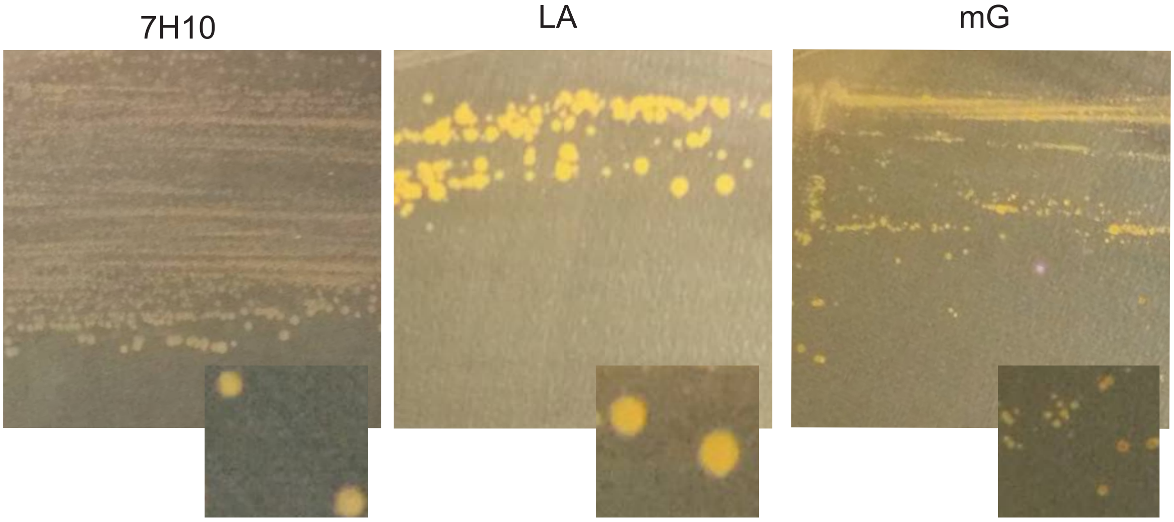

Figure S2

Growth curves for *Mmon*^{T} and *Mboe*^{T}.

(A) Growth curve for *Mmon*^{RFPHyg} in liquid mG media supplemented with hygromycin (100 μg ml^{-1})

(B) Growth curve for *Mboe*^{T} cultivated in liquid 7H9 medium. Generation times (GTs) were determined to be ≈3 hours (Expo I) and ≈11.5 hours (Expo II). The time points considered for each exponential phase (Expo I and Expo II) are marked in red.

(C) Bi-phasic growth curve for *Mboe*^{T} where the red arrow indicates the time point in the Expo II phase when the cells were subjected to the different conditions as described in the main text Section 2.

Fig S2

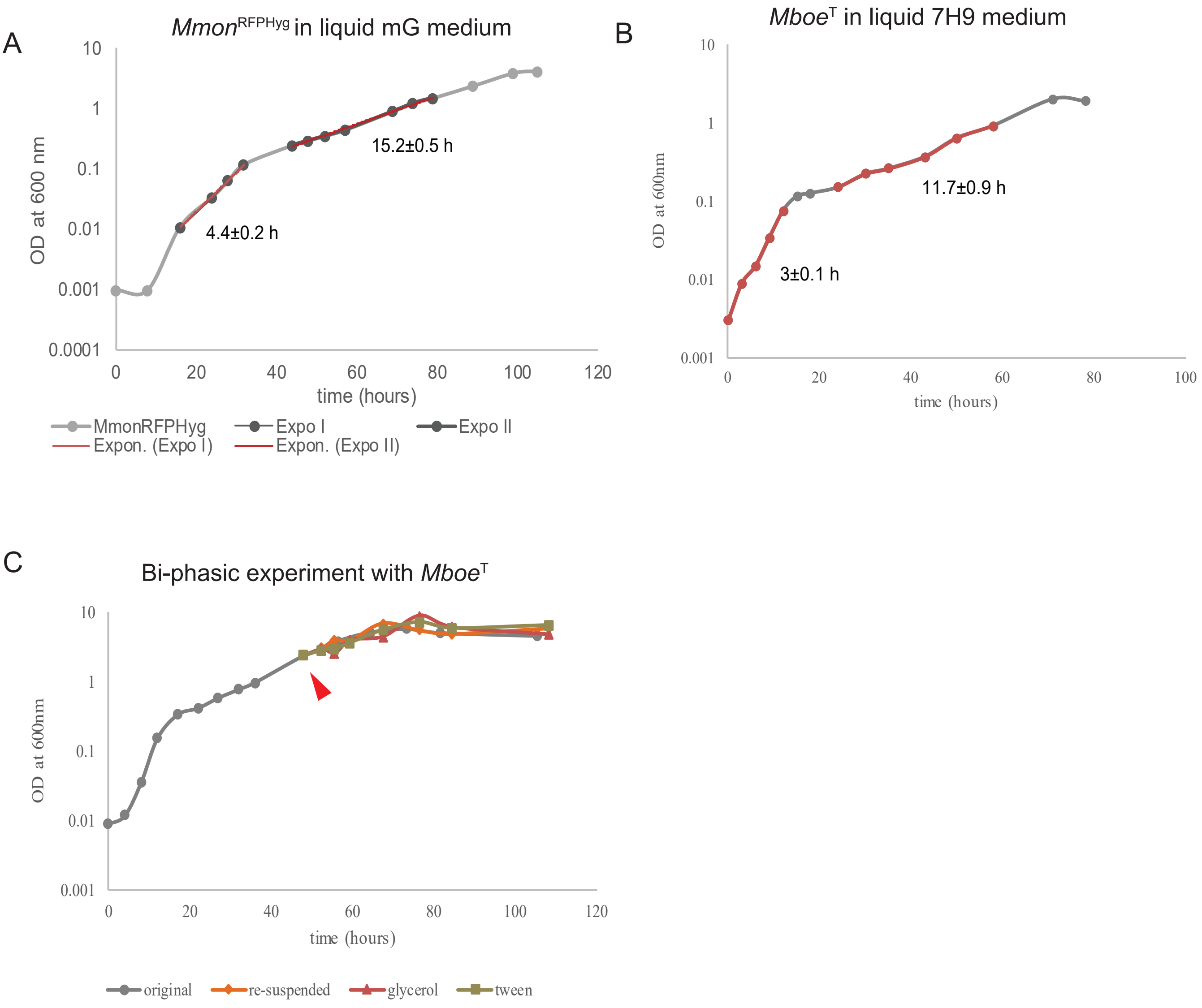

A
Mmon^RFPHyg in liquid mG medium
OD at 600 nm
10
1
0.1
0.01
0.001
0.0001
0
20
40
60
80
100
120
time (hours)
4.4±0.2 h
15.2±0.5 h
MmonRFPHyg
Expo I
Expo II
Expon. (Expo I)
Expon. (Expo II)
B
Mboe^T in liquid 7H9 medium
OD at 600nm
10
1
0.1
0.01
0.001
0
20
40
60
80
100
time (hours)
3±0.1 h
11.7±0.9 h
C
Bi-phasic experiment with Mboe^T
OD at 600nm
10
1
0.1
0.01
0.001
0
20
40
60
80
100
120
time (hours)
original
re-suspended
glycerol
tween

Figure S3

Classification of observed cell morphologies.

(A) Different cell morphologies observed and considered in this study- rods (average length of 1.8±0.31 μm), coccoid (with average diameter of 0.8±0.19 μm) and PGB cells marked with a yellow arrow. White scale bars = 1 μm.

(B) Different growth phases of *Mmon*^{RFPHyg} on solid 7H10 medium.

Fig S3

A

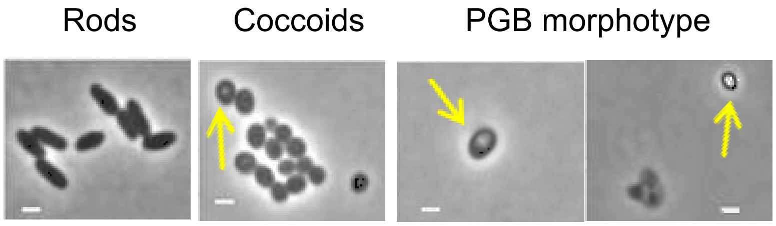
Rods
Coccoids
PGB morphotype

B

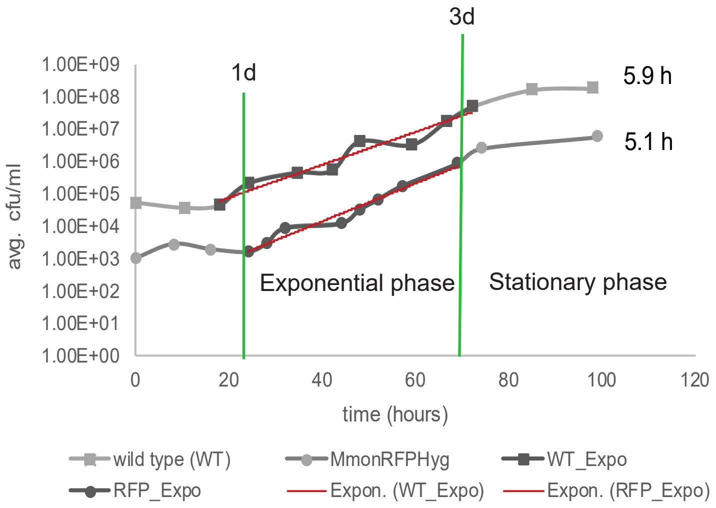
3d
1d
1.00E+09
1.00E+08
1.00E+07
1.00E+06
1.00E+05
1.00E+04
1.00E+03
1.00E+02
1.00E+01
1.00E+00
avg. cfu/ml
5.9 h
5.1 h
Exponential phase
Stationary phase
0
20
40
60
80
100
120
time (hours)
wild type (WT)
MmonRFPHyg
WT_Expo
RFP_Expo
Expon. (WT_Expo)
Expon. (RFP_Expo)

Figure S4

Change in cell morphology in *Mmon*^{T} and *Mboe*^{T} cultures.

(A) Average percentage distribution of *Mmon*^{T} cell morphologies on mG plates at different time points as indicated (d = time expressed in days).

(B) Average percentage distribution of *Mmon*^{RFPHyg} cell morphologies on mG plates supplemented with hygromycin (100 µg mL^{-1}) at different time points as indicated (d = time expressed in days).

(C) Time course microscopy for *Mmon*^{RFPHyg} grown in 7H9 liquid medium supplemented with hygromycin (100 µg mL^{-1}) illustrating changes in cell shape and appearance of spore-like cells (yellow arrows). White scale bars = 2 µm.

(D) Time course microscopy for *Mboe*^{T} grown on 7H10 plates. Yellow arrows mark spore-like cells. White scale bars = 1 µm.

(E) Average cell size of *Mboe*^{T} cell morphologies in liquid 7H9 media at different time points as indicated.

(F) Shows the time points chosen for transcriptome and its corresponding frequencies of the different cell morphologies.

(G) Microscopy images of re-grown cultures (incubated for 5 days on plates) of *Mmon*^{RFPHyg} re-streaked from 50 days old cultures (colonies from plates), stained with DAPI (blue) and MTG (green). Bottom panel shows microscopy images of re-grown cultures (on plates) of *Mmon*^{T} from 60 days old cultures (from plates), stained with FM4-64 (red), DAPI (blue) and MTG (green). White scale bars = 2 µm.

Fig S4

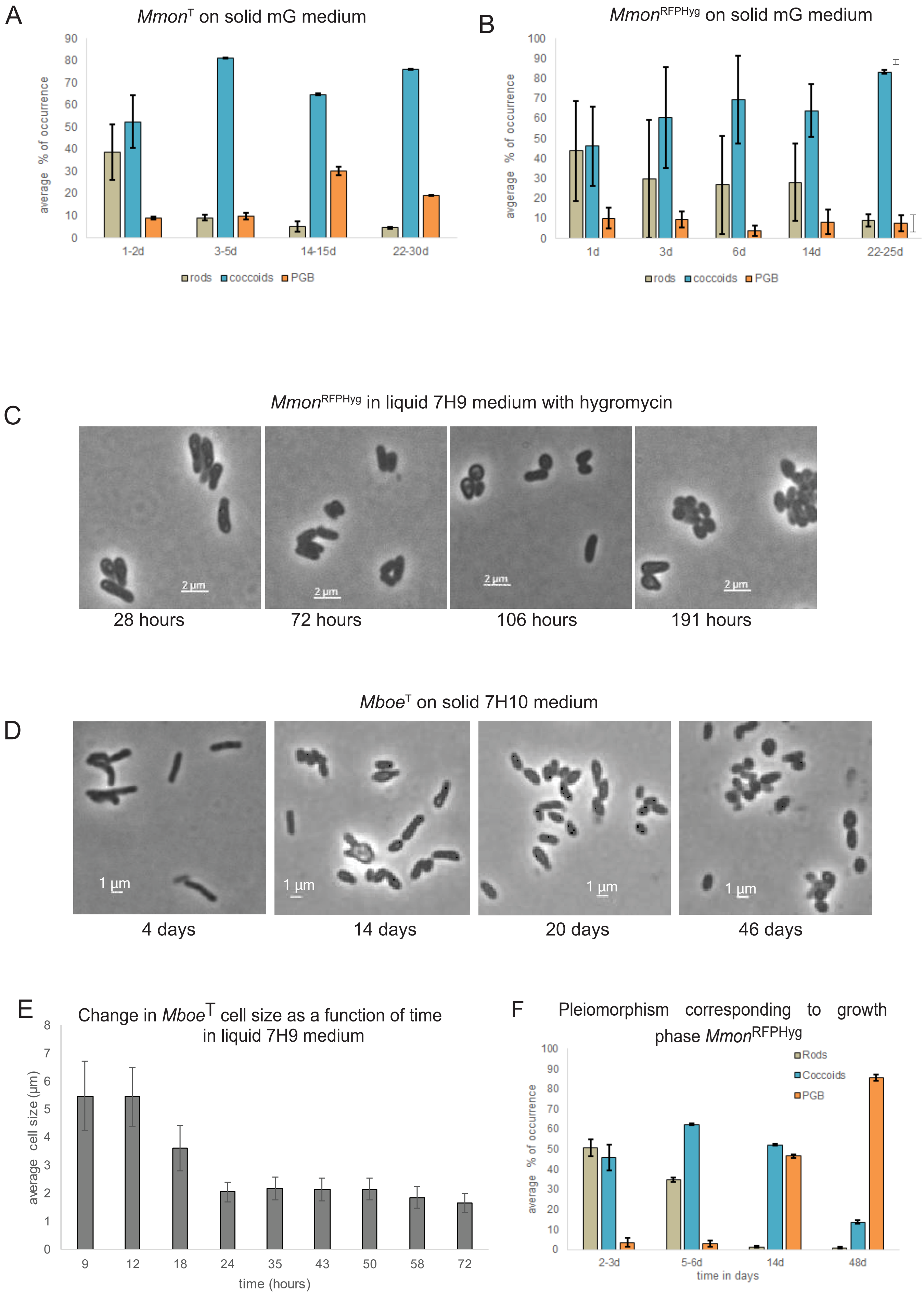
A
Mmon^T on solid mG medium
average % of occurrence
90
80
70
60
50
40
30
20
10
0
1-2d
3-5d
14-15d
22-30d
rods
coccoids
PGB
B
Mmon^RFPHyg on solid mG medium
average % of occurrence
100
90
80
70
60
50
40
30
20
10
0
1d
3d
6d
14d
22-25d
rods
coccoids
PGB
C
Mmon^RFPHyg in liquid 7H9 medium with hygromycin
2 µm
2 µm
2 µm
2 µm
28 hours
72 hours
106 hours
191 hours
Mboe^T on solid 7H10 medium
D
1 µm
1 µm
1 µm
1 µm
4 days
14 days
20 days
46 days
E
Change in Mboe^T cell size as a function of time in liquid 7H9 medium
average cell size (µm)
8
7
6
5
4
3
2
1
0
9
12
18
24
35
43
50
58
72
time (hours)
F
Pleiomorphism corresponding to growth phase Mmon^RFPHyg
average % of occurrence
100
90
80
70
60
50
40
30
20
10
0
Rods
Coccoids
PGB
2-3d
5-6d
14d
48d
time in days

G

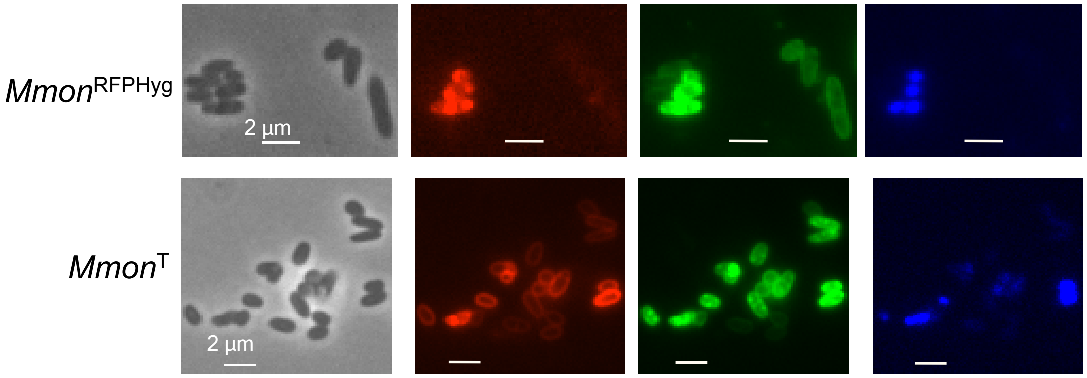

Figure S5

Differential expression and percentage distribution of Serine Threonine kinases (STPKs) in *Mmon*.

(A-C) Change expressed as log_2-fold for STPK mRNA levels at 6, 14 and 48 days relative to the levels at 3 days, where a negative log_2-value indicates higher level in exponentially growing cells. Statistical significance, see main text Section 2, *$p<0.05$; **$p<0.01$; ***$p<0.001$.

(D-G) Distribution of STPK mRNA levels in (100% correspond to the sum of all STPK mRNAs):

(D) Exponentially growing *Mmon*^{RFPHyg} cells (3 days),

(E) 6 days old *Mmon*^{RFPHyg} cells,

(F) 14 days old *Mmon*^{RFPHyg} cells and

(G) 48 days old *Mmon*^{RFPHyg} cells.

According to the annotation the STPKs marked in grey lack the kinase domain.

Fig S5

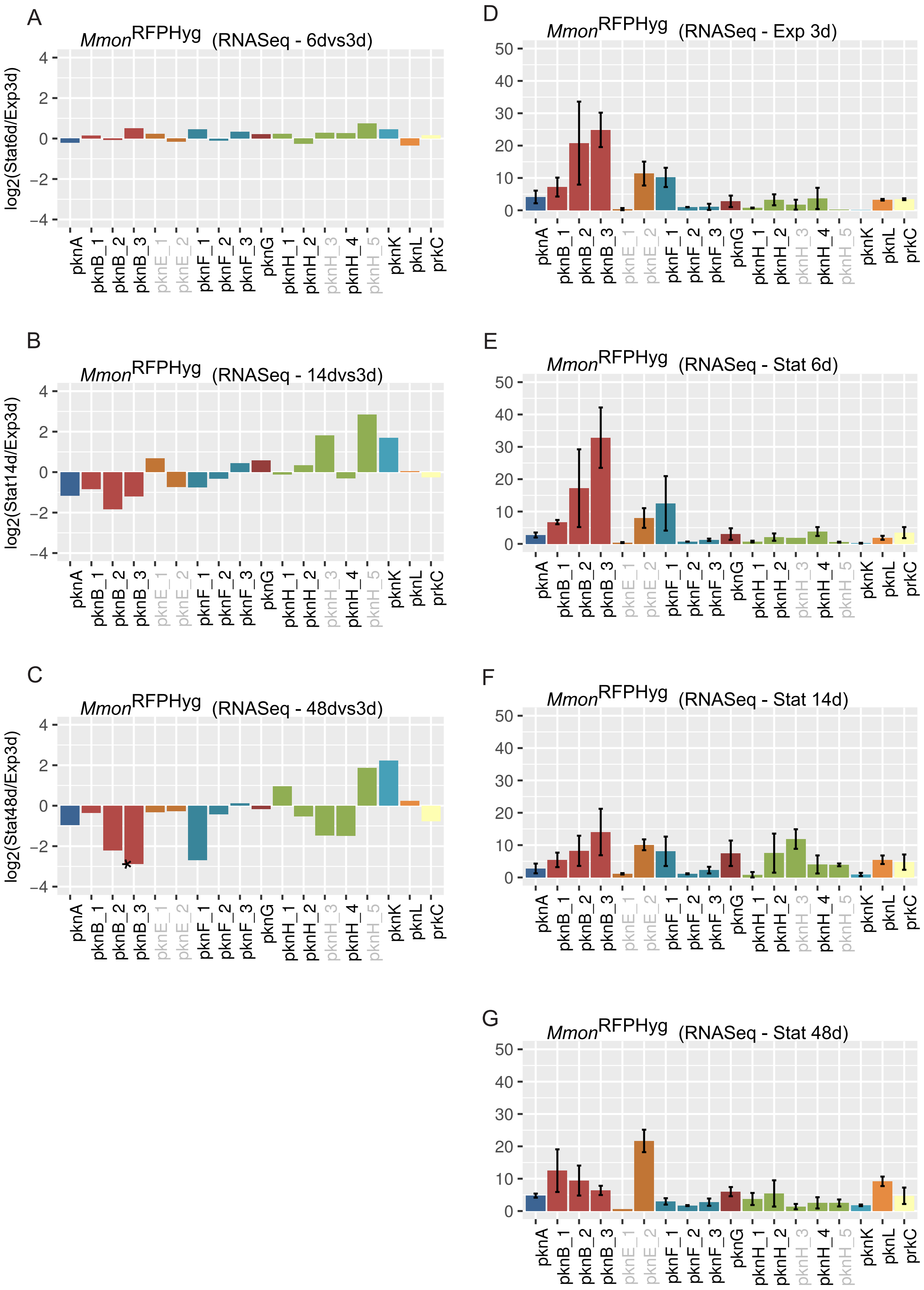
A
MmonRFPHyg (RNASeq - 6dvs3d)
log2(Stat6d/Exp3d)
4
2
0
−2
−4
B
MmonRFPHyg (RNASeq - 14dvs3d)
log2(Stat14d/Exp3d)
C
MmonRFPHyg (RNASeq - 48dvs3d)
log2(Stat48d/Exp3d)
*
D
MmonRFPHyg (RNASeq - Exp 3d)
E
MmonRFPHyg (RNASeq - Stat 6d)
F
MmonRFPHyg (RNASeq - Stat 14d)
G
MmonRFPHyg (RNASeq - Stat 48d)
50
40
30
20
10
0
pknA
pknB_1
pknB_2
pknB_3
pknE_1
pknE_2
pknF_1
pknF_2
pknF_3
pknG
pknH_1
pknH_2
pknH_3
pknH_4
pknH_5
pknK
pknL
prkC

Figure S6

*dnaK*_3 analysis: protein domain architecture, controls for the observed morphology changes (statistics).

(A) Predicted protein domain architecture of DnaK protein using SMARTdb tool (see main text, Section 2). The presence of the MreB_Mbl domain architecture as part of the DnaK protein in indicated bacteria are shown with the corresponding protein locus tags.

(B) Bar plots showing distribution (expressed in percentage) of the different morphologies in *Mmar*$^{\text{pBS401-antidnaK3Mmon}}$ (increased number of genes and induction).

(C) Average occurrence of the different cell morphologies in *Mmar*^{RFPHyg} and *Mmar*$^{\text{pBS401}}$ (with and without tetracycline induction; see main text, Section 2): controls for experiments shown in Figure 6. White scale bars = 1 μm.

Fig S6

A

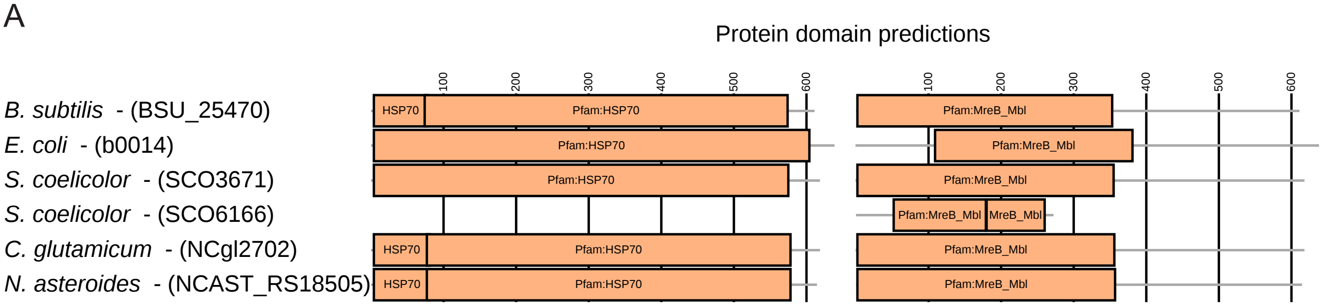
Protein domain predictions
B. subtilis - (BSU_25470)
E. coli - (b0014)
S. coelicolor - (SCO3671)
S. coelicolor - (SCO6166)
C. glutamicum - (NCgl2702)
N. asteroides - (NCAST_RS18505)
HSP70
Pfam:HSP70
Pfam:MreB_Mbl
MreB_Mbl
100
200
300
400
500
600

B

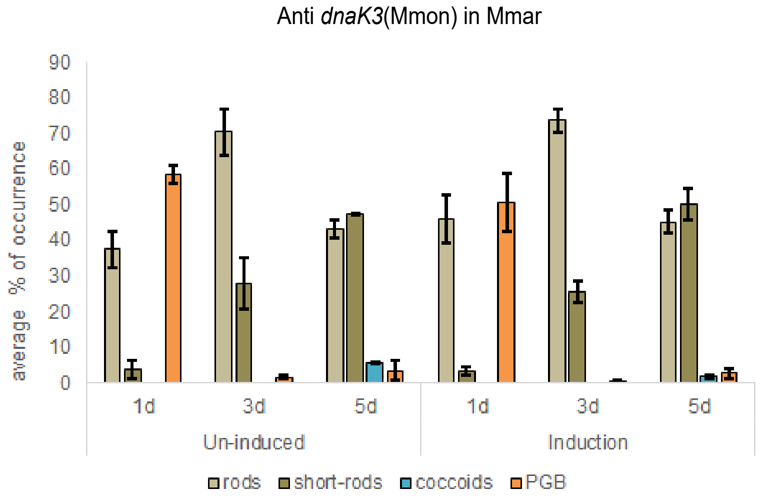
Anti dnaK3(Mmon) in Mmar
average % of occurrence
0
10
20
30
40
50
60
70
80
90
1d
3d
5d
Un-induced
Induction
rods
short-rods
coccoids
PGB

C

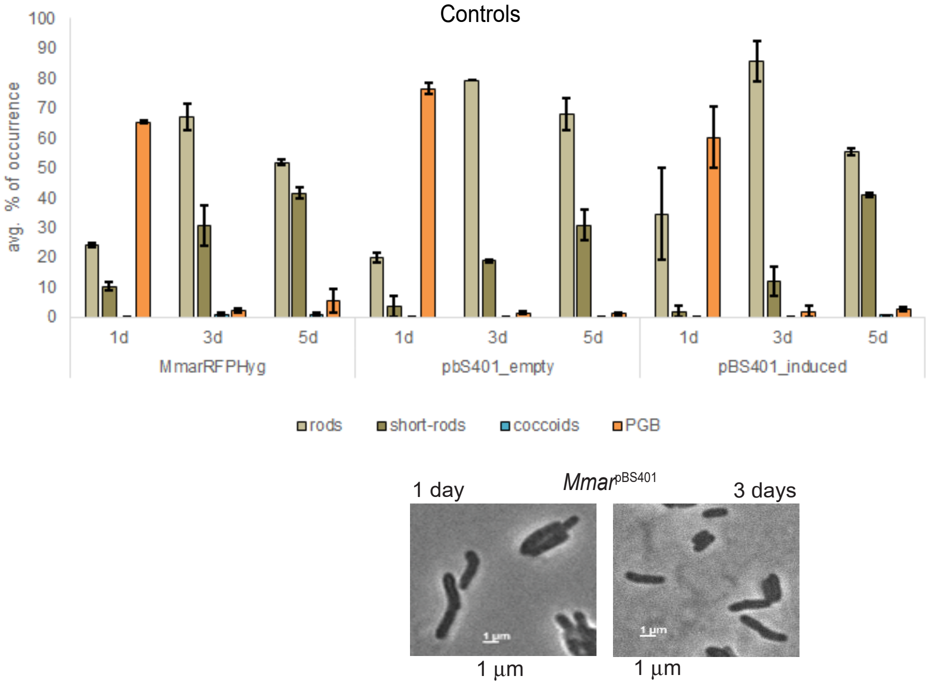
Controls
avg. % of occurrence
0
10
20
30
40
50
60
70
80
90
100
1d
3d
5d
MmarRFPHyg
pbS401_empty
pBS401_induced
rods
short-rods
coccoids
PGB
1 day
Mmar pBS401
3 days
1 µm
1 µm

Figure S7

Analysis of the *dnaK*_3 regulatory region.

Comparison of upstream of *dnaK*_3 in *Mmon*^{T} and *Mmar*^{T} with the *dnaK* region in *Mtb*^{H37Rv}, 300 nucleotides upstream of *dnaK*_3 gene was considered and boxes/nucleotides marked with different colours to represent putative promoters, and predicted HAIR, CRP, RegX3 and MtrA binding sites as indicated. Overlapping promoter sequences are marked with respective coloured lines while nucleotides in green mark the predicted Shine-Dalgarno sequences upstream of the putative translational start site, ATG is marked in red.

Fig S7

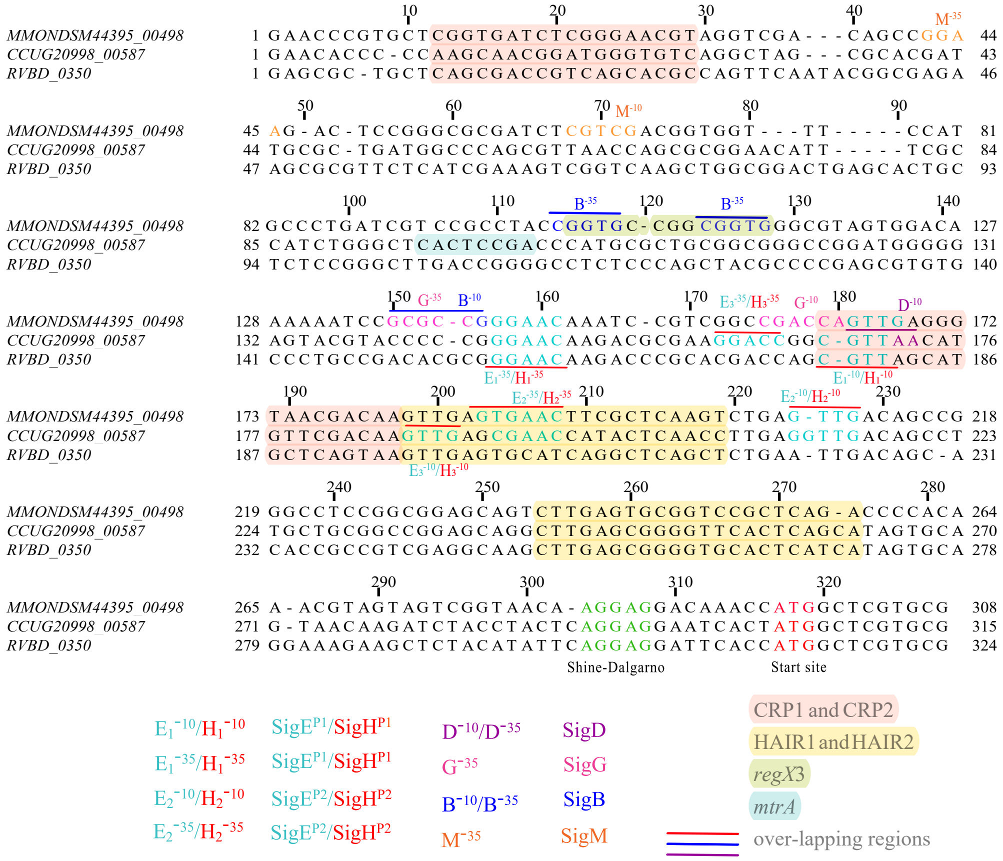

MMONDSM44395_00498 1 GAACCCGTGCTCGGTGATCTCGGGAACGTAGGTCGA---CAGCCGGA 44
CCUG20998_00587 1 GAACACCC-CCAAGCAACGGATGGGTGTCAGGCTAG---CGCACGAT 43
RVBD_0350 1 GAGCGC-TGCTCAGCGACCGTCAGCACGCCAGTTCAATACGGCGAGA 46
MMONDSM44395_00498 45 AG-AC-TCCGGGCGCGATCTCGTCGACGGTGGT---TT-----CCAT 81
CCUG20998_00587 44 TGCGC-TGATGGCCCAGCGTTAACCAGCGCGGAACATT-----TCGC 84
RVBD_0350 47 AGCGCGTTCTCATCGAAAGTCGGTCAAGCTGGCGGACTGAGCACTGC 93
MMONDSM44395_00498 82 GCCCTGATCGT CCGCCTACCGGTGC-CGGCGGTGGGCGTAGTGGACA 127
CCUG20998_00587 85 CATCTGGGCTCACTCCGACCCATGCGCTGCGGCGGGCCGGATGGGGG 131
RVBD_0350 94 TCTCCGGGCTTGACCGGGGCCTCTCCCAGCTACGCCCCGAGCGTGTG 140
MMONDSM44395_00498 128 AAAAATCCGCGC-CGGGAACAAATC-CGTCGGCCGACCAGTTGAGGG 172
CCUG20998_00587 132 AGTACGTACCCC-CGGGAACAAGACGCGAAGGACCGGC-GTTAACAT 176
RVBD_0350 141 CCCTGCCGACACGCGGGAACAAGACCCGCACGACCAGC-GTTAGCAT 186
MMONDSM44395_00498 173 TAACGACAAGTTGAGTGAACTTCGCTCAAGTCTGAG-TTGACAGCCG 218
CCUG20998_00587 177 GTTCGACAAGTTGAGCGAACCATACTCAACCTTGAGGTTGACAGCCT 223
RVBD_0350 187 GCTCAGTAAGTTGAGTGCATCAGGCTCAGCTCTGAA-TTGACAGC-A 231
MMONDSM44395_00498 219 GGCCTCCGGCGGAGCAGTCTTGAGTGCGGTCCGCTCAG-ACCCCACA 264
CCUG20998_00587 224 TGCTGCGGCCGGAGCAGGCTTGAGCGGGGTTCACTCAGCATAGTGCA 270
RVBD_0350 232 CACCGCCGTCGAGGCAAGCTTGAGCGGGGTGCACTCATCATAGTGCA 278
MMONDSM44395_00498 265 A-ACGTAGTAGTCGGTAACA-AGGAGGACAAACCATGGCTCGTGCG 308
CCUG20998_00587 271 G-TAACAAGATCTACCTACTCAGGAGGAATCACTATGGCTCGTGCG 315
RVBD_0350 279 GGAAAGAAGCTCTACATATTCAGGAGGATTCACCATGGCTCGTGCG 324
Shine-Dalgarno
Start site
E1-10/H1-10 SigEP1/SigHP1
E1-35/H1-35 SigEP1/SigHP1
E2-10/H2-10 SigEP2/SigHP2
E2-35/H2-35 SigEP2/SigHP2
D-10/D-35 SigD
G-35 SigG
B-10/B-35 SigB
M-35 SigM
CRP1 and CRP2
HAIR1 and HAIR2
regX3
mtrA
over-lapping regions

Figure S8

Differential mRNA levels of *regX*3 and *senX*3 in *Mmon*^{RFPHyg} and *Mmar*^{RFPHyg}.

Fig S8

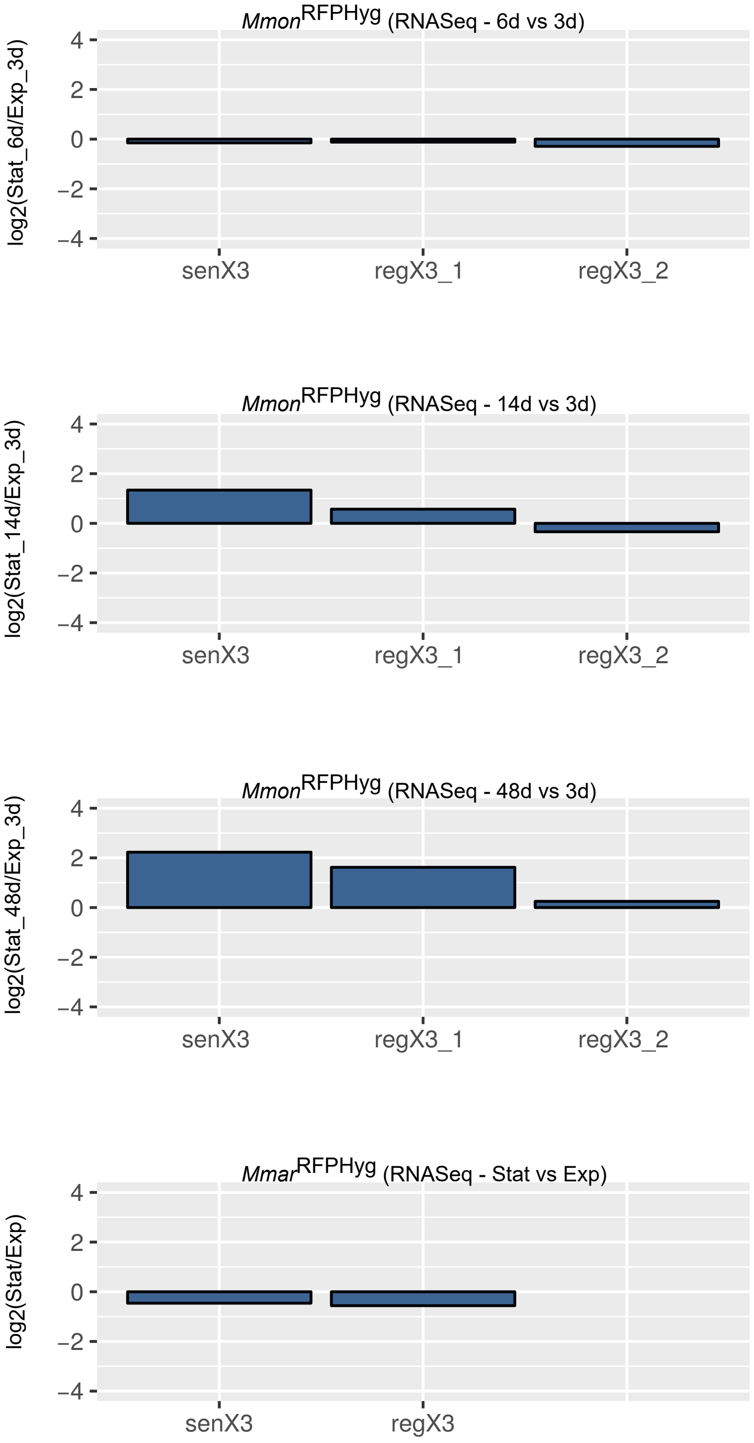

*Mmon*RFPHyg (RNASeq - 6d vs 3d)
log2(Stat_6d/Exp_3d)
senX3
regX3_1
regX3_2
*Mmon*RFPHyg (RNASeq - 14d vs 3d)
log2(Stat_14d/Exp_3d)
*Mmon*RFPHyg (RNASeq - 48d vs 3d)
log2(Stat_48d/Exp_3d)
*Mmar*RFPHyg (RNASeq - Stat vs Exp)
log2(Stat/Exp)
regX3

Figure S9

Sigma factor mRNA levels in *Mmon*^{RFPHyg} and *Mmar*^{RFPHyg} as a function of growth on 7H10 plates at 37°C as determined by RNASeq (note that panels H-J correspond to Figs S5A-C in Ref [67] and were included for comparison, see also [41]).

(A-C) Change expressed as $\log_2$-fold change for sigma factor mRNA levels at 6, 14 and 48 days relative to the levels at 3 days as indicated, where a negative $\log_2$-value indicates higher level in exponential growing cells. Statistical significance, see main text Section 2, *$p<0.05$; **$p<0.01$; ***$p<0.001$.

(D-G) Distribution of sigma factor mRNA levels in (100% correspond to the sum for all sigma factor mRNAs):

(D) exponentially growing *Mmon*^{RFPHyg} cells (3 days),

(E) 6 days old *Mmon*^{RFPHyg} cells,

(F) 14 days old *Mmon*^{RFPHyg} cells and

(G) 48 days old *Mmon*^{RFPHyg} cells.

(H) Change in *Mmar*^{RFPHyg} sigma factor mRNA levels in stationary phase relative to the levels in exponentially growing cells expressed as $\log_2$-fold change. Negative $\log_2$-value indicate higher level in exponential growing cells. Statistical significance, see main text Section 2, *$p<0.05$; **$p<0.01$; ***$p<0.001$.

(I-J) Distribution of sigma factor mRNA levels in (100% correspond to the sum of all sigma factor mRNAs):

I) Exponentially growing *Mmar*^{RFPHyg} cells, $OD_{600} \approx 0.5$.

J) Stationary *Mmar*^{RFPHyg} cells, $OD_{600} \approx 4.5$.

Fig S9

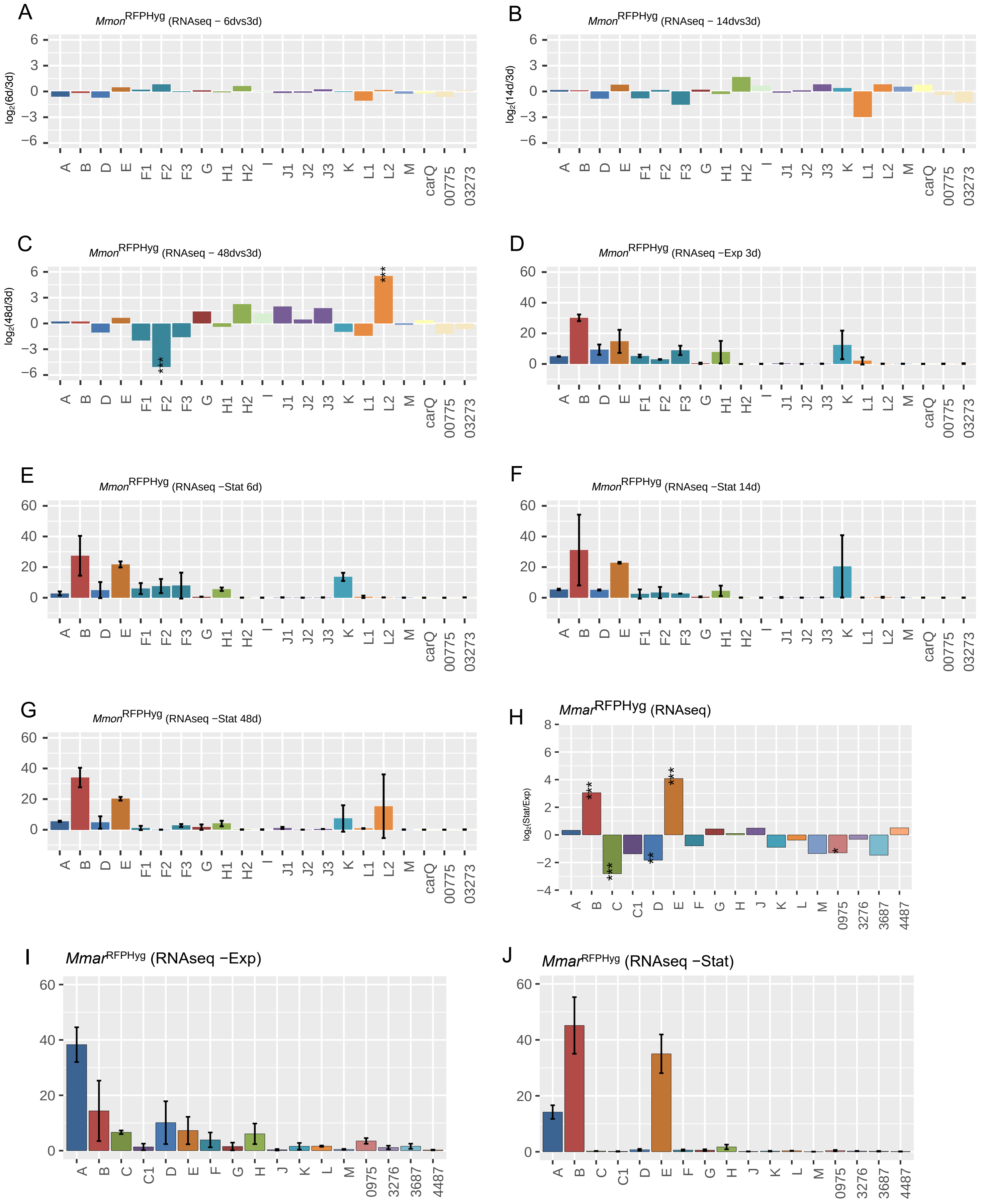
A
$Mmon^{RFPHyg}$ (RNAseq – 6dvs3d)
log₂(6d/3d)
B
$Mmon^{RFPHyg}$ (RNAseq – 14dvs3d)
log₂(14d/3d)
C
$Mmon^{RFPHyg}$ (RNAseq – 48dvs3d)
log₂(48d/3d)
D
$Mmon^{RFPHyg}$ (RNAseq –Exp 3d)
E
$Mmon^{RFPHyg}$ (RNAseq –Stat 6d)
F
$Mmon^{RFPHyg}$ (RNAseq –Stat 14d)
G
$Mmon^{RFPHyg}$ (RNAseq –Stat 48d)
A B D E F1 F2 F3 G H1 H2 I J1 J2 J3 K L1 L2 M carQ 00775 03273
H
$Mmar^{RFPHyg}$ (RNAseq)
log₂(Stat/Exp)
I
$Mmar^{RFPHyg}$ (RNAseq –Exp)
J
$Mmar^{RFPHyg}$ (RNAseq –Stat)
A B C C1 D E F G H J K L M 0975 3276 3687 4487

Figure S10

Genome-wide distribution of sigma factor genes in *Mmon*^{T} and *Mmar*^{T}.

Circos plot of pair-wise genome-wide distribution of sigma factor genes in *Mmon*^{T} and *Mmar*^{T}. The curves inside the plot connect orthologs between the two genomes. Generation of circus plot, see http://circos.ca.

Fig S10

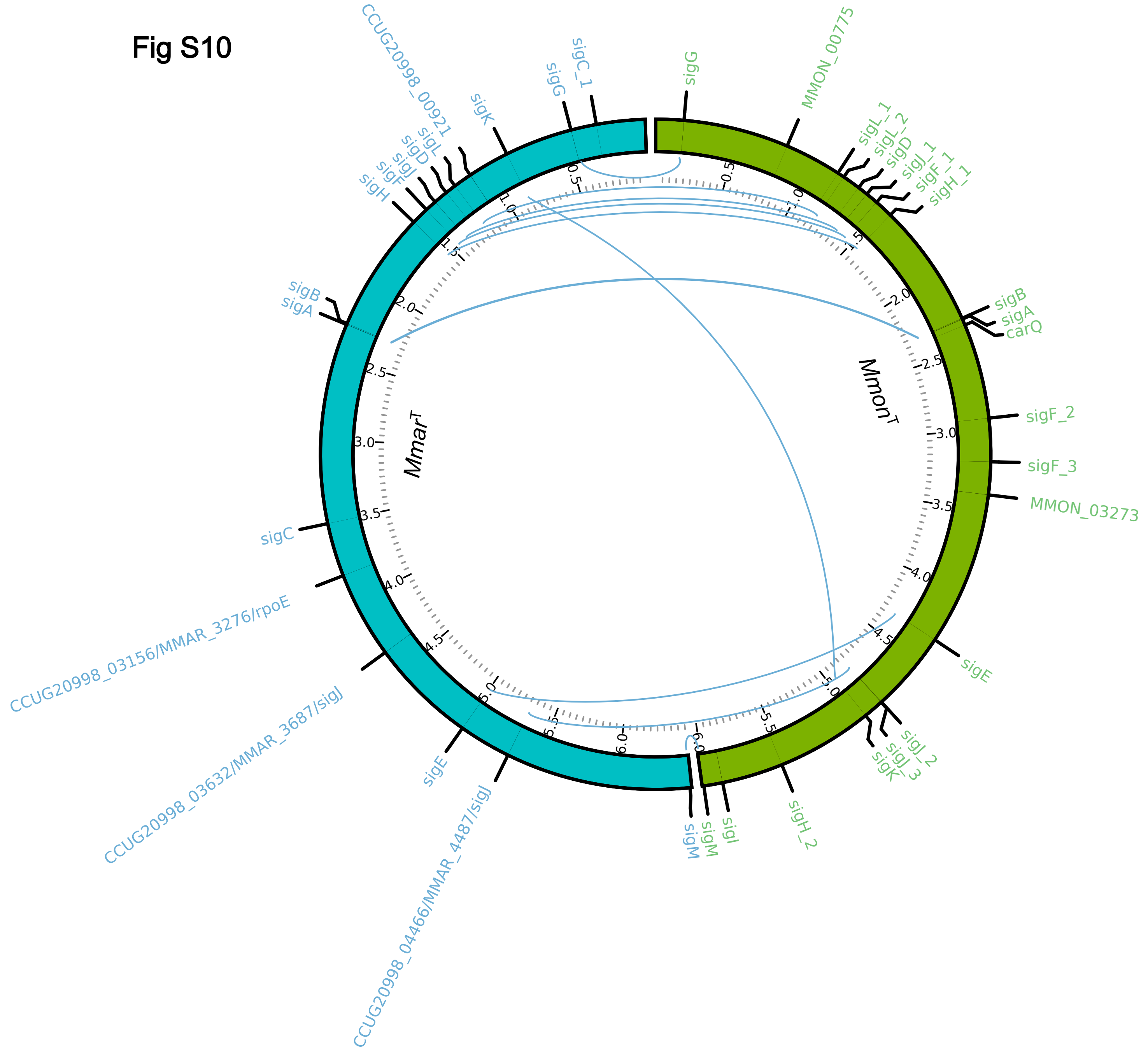
CCUG20998_00921
sigC_1
sigG
sigK
sigL
sigD
sigJ
sigF
sigH
sigB
sigA
sigC
CCUG20998_03156/MMAR_3276/rpoE
CCUG20998_03632/MMAR_3687/sigJ
sigE
CCUG20998_04466/MMAR_4487/sigJ
sigM
sigG
MMON_00775
sigL_1
sigL_2
sigD
sigJ_1
sigF_1
sigH_1
sigB
sigA
carQ
sigF_2
sigF_3
MMON_03273
sigE
sigJ_2
sigJ_3
sigK
sigM
sigI
sigH_2
MmarT
MmonT

Figure S11

Microscopy images of *Mmon* (28 days old) cells stained with Malachite (green) and Safranin (red).

The refractive cells appeared both red and green (and overlay in yellow; see supplementary Material and Methods). White scale bars = 2 µm.

Fig S11

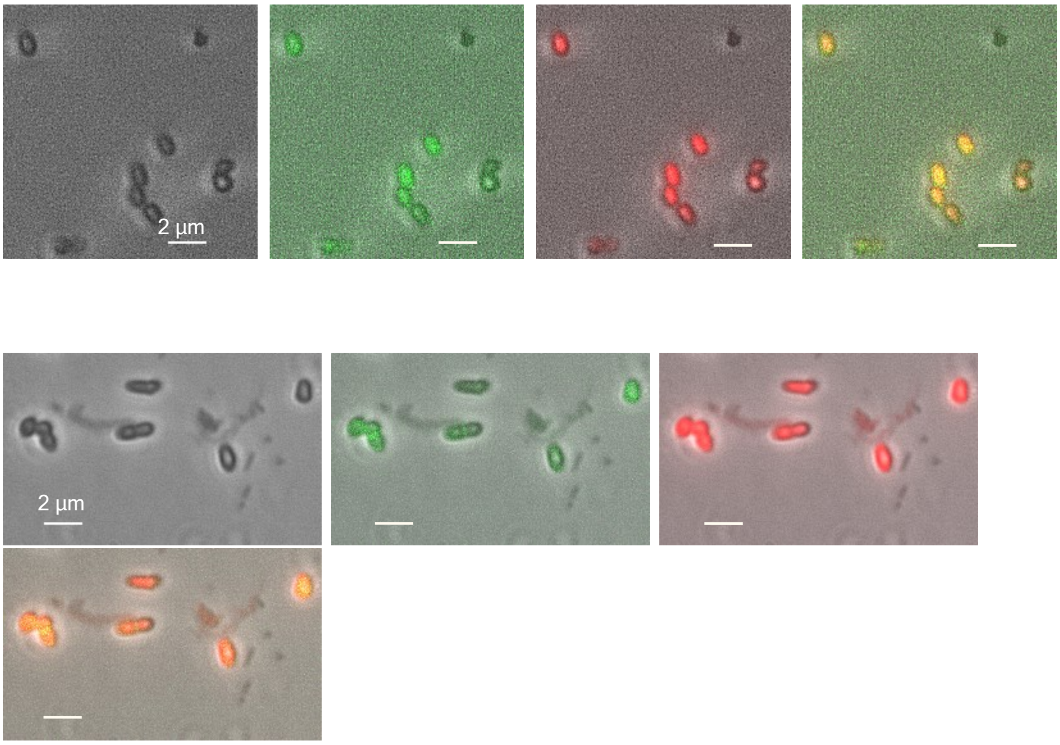

Figure S12

DAPI staining of *Mmon*^{T} cells.

(A) 28 days old *Mmon*^{T} stained with DAPI (blue), MTG (green) and FM4-64 (red). Refractive PGB cells showed DAPI staining around the refractive regions (white arrows). White scale bars = 2 μm.

(B) Roughly two weeks old *Mmon*^{T} cells incubated at 37°C for 15-30 min post staining slightly enhanced the DAPI staining efficiency of the refractive regions. White scale bars = 2 μm.

Fig S12

A

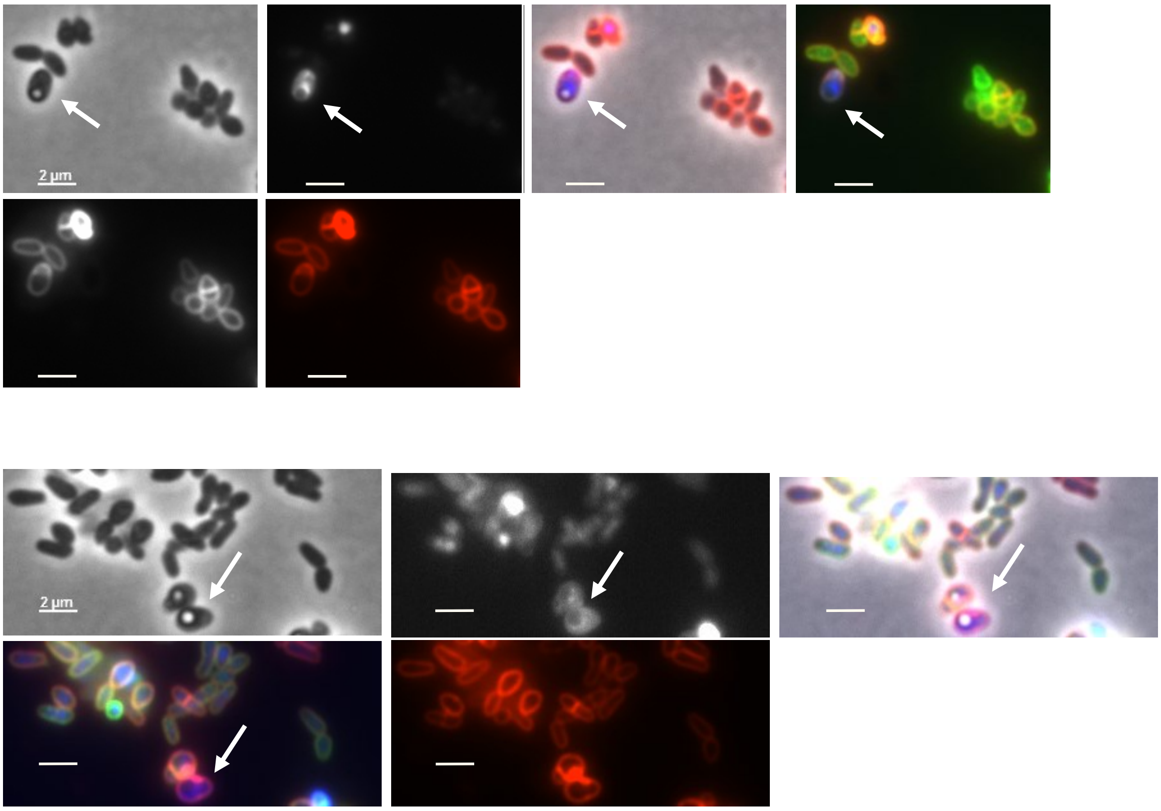

B

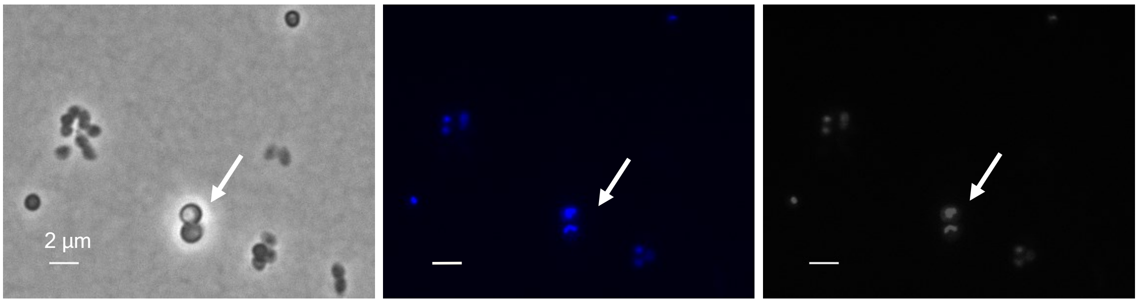

Supplement: Supplementary file 1 [file microorganisms-13-00475-s001.zip › microorganisms-3391607-supplementary.pdf]
